# rs4143815-*PDL1*, a New Potential Immunogenetic Biomarker of Biochemical Recurrence in Locally Advanced Prostate Cancer after Radiotherapy

**DOI:** 10.3390/ijms20092082

**Published:** 2019-04-27

**Authors:** Chiara Zanusso, Eva Dreussi, Roberto Bortolus, Chiara Romualdi, Sara Gagno, Elena De Mattia, Loredana Romanato, Franca Sartor, Luca Quartuccio, Erika Cecchin, Giuseppe Toffoli

**Affiliations:** 1 Experimental and Clinical Pharmacology, Centro di Riferimento Oncologico di Aviano (CRO), IRCCS, 33081 Aviano, Italy; czanusso@cro.it (C.Z.); edreussi@cro.it (E.D.); sgagno@cro.it (S.G.); edemattia@cro.it (E.D.M.); lromanato@cro.it (L.R.); fsartor@cro.it (F.S.); ececchin@cro.it (E.C.); 2 Department of Radiation Oncology, Centro di Riferimento Oncologico di Aviano (CRO), IRCCS, 33081 Aviano, Italy; rbortolus@cro.it; 3 Department of Biology, University of Padova, 35122 Padova, Italy; chiara.romualdi@unipd.it; 4 Rheumatology Clinic, Department of Medical and Biological Sciences, University Hospital “Santa Maria della Misericordia”, 33100 Udine, Italy; luca.quartuccio@asuiud.sanita.fvg.it

**Keywords:** prostate cancer, immunogenetics, biomarker, radiotherapy, biochemical recurrence

## Abstract

Up to 30–50% of patients with locally advanced prostate cancer (PCa) undergoing radiotherapy (RT) experience biochemical recurrence (BCR). The immune system affects the RT response. Immunogenetics could define new biomarkers for personalization of PCa patients’ treatment. The aim of this study is to define the immunogenetic biomarkers of 10 year BCR (primary aim), 10 year overall survival (OS) and 5 year BCR (secondary aims). In this mono-institutional retrospective study, 549 Caucasian patients (a discovery set *n* = 418; a replication set *n* = 131) were affected by locally advanced PCa and homogeneously treated with RT. In the training set, associations were made between 447 SNPs in 77 genes of the immune system; and 10 year BCR and 10 year OS were tested through a multivariate Cox proportional hazard model. Significant SNPs (*p*-value < 0.05, *q*-value < 0.15) were analyzed in the replication set. Replicated SNPs were tested for 5 year BCR in both sets of patients. A polymorphism in the *PDL1* gene (rs4143815) was the unique potential genetic variant of 10 year BCR (training set: *p* = 0.003, HR (95% CI) = 0.58 (0.41–0.83); replication set: *p* = 0.063, HR (95% CI) = 0.52 (0.26–1.04)) that was significantly associated with 5 year BCR (training set: *p* = 0.009, HR (95% CI) = 0.59 (0.40–0.88); replication set: *p* = 0.036, HR (95% CI) = 0.39 (0.16–0.94)). No biomarkers of OS were replicated. rs4143815-*PDL1* arose as a new immunogenetic biomarker of BCR in PCa, giving new insights into the RT/immune system interaction, which could be potentially useful in new approaches using anti-PDL1 therapies for PCa.

## 1. Introduction

Prostate cancer (PCa) is one of the current major health problems, considering its high prevalence. Radical radiotherapy (RT), either alone or in combination with hormone therapy (HT), is the treatment of choice for locally advanced PCa. Nonetheless, a non-negligible percentage of patients relapse, with biochemical recurrence (BCR) being detected in 30–50% of patients. BCR is closely connected with metastasis free survival (MFS), a strong surrogate for overall survival (OS) in clinically localized PCa [1,2].

The definition of markers that identify patients with a higher risk of relapse could be implemented through more frequent follow-ups and in more appropriate maintenance therapy.

RT exerts not only a local effect at the tumor level, but it fosters the activity of the host immune system both in the tumor microenvironment and at the systemic level. In this scenario, immunogenetics, the study of polymorphisms (SNPs) in genes codifying for factors involved in immune system activity and maturation, represents one way to better elucidate the role of the immune system in patients’ outcomes after RT and in the definition of biomarkers of possible clinical interest. Indeed, SNPs located in genes involved in host immune systems (i.e., *RNASEL*, *TGF-β*, *Bcl2*) have been associated with treatment response, in terms of toxicity and efficacy [3,4], and patients’ prognosis [5,6].

Biomarkers of the RT response could be potentially used in the definition of patients possibly eligible for new treatments such as radioimmunotherapy [7]. Currently, immunotherapy, either alone or in association with chemotherapy, is entered into the guidelines of different cancer treatments, and the association of immunotherapy with RT represents a new challenge. In PCa, data about the role of RT in association with immunotherapy are still too scarce to draw any final conclusions [8], but the deepening of these mechanisms could have a clinical impact.

On these grounds, the main aim of this study was to define, with an immunogenetics approach, new potential biomarkers of 10 year BCR in locally advanced PCa patients undergoing radical RT. The secondary aims were the definition of biomarkers of 10 year OS and 5 year BCR. The potential prognostic role of 447 SNPs in 77 genes, for both 10 year BCR and 10 year OS, were tested in a discovery set (*n* = 418 patients). The significant associations determined in this step were then verified in the replication set (*n* = 131 patients). Only replicated SNPs were tested for 5 year BCR.

## 2. Results

### 2.1. Patient Characteristics and Genotyping

Patients’ clinical and pathological data were collected from the medical records. The discovery and the replication set were well balanced for tumor grade, age, Gleason score, PSA levels, D’Amico risk groups, and treatment parameters (Table 1).

In the training set, most genetic analyses (331/447 assays—74.05%) had a genotype call rate of higher than 95.00% (range: 76.32–100.00%). In the replication set, genetic analyses had an average genotype call rate of 97.26% (range: 75.57–100.00%).

### 2.2. Associations of SNPs with 10 Year BCR

In the training set, PSA levels at diagnosis, age, Gleason score, hormone therapy, and tumor grade were strongly associated with 10 year BCR and were used as covariates for multivariate analysis. Nineteen SNPs were significantly associated with 10 year BCR (*p* < 0.05 and *q* < 0.15) (Table 2).

**Figure 1:**
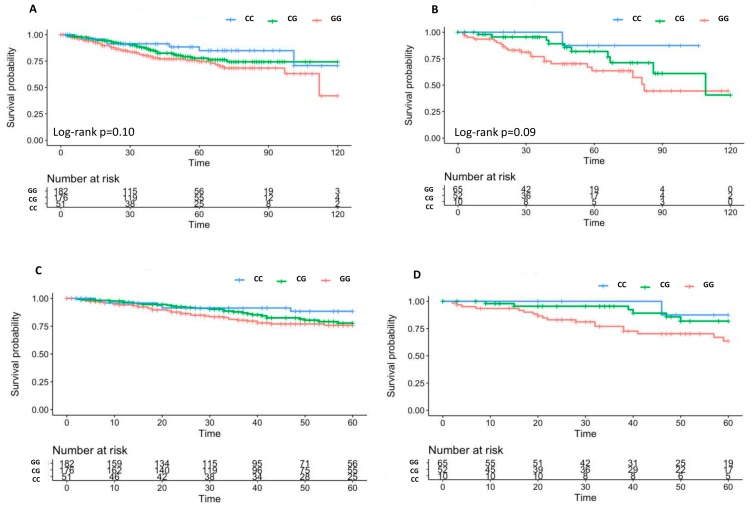
Kaplan–Meier curves of 10 year BCR according to rs4143815-*PDL1* in the training set (**A**) and in the replication set (**B**) and Kaplan–Meier curves of 5 year BCR after RT according to rs4143815-*PDL1* in the training set (**C**) and in the replication set (**D**).

The association between rs4143815-*PDL1* and 5 year BCR was tested. Interestingly, significant associations were found both in the training set (*p* = 0.009, HR (95% CI) = 0.59 (0.40–0.88) and replication set (*p* = 0.036, HR (95% CI) = 0.39 (0.16–0.94)) (Figure 1). However, the association between rs1411262-*PDL1* and 5 year BCR was significant only in the training set (Supplementary Figure S1).

### 2.3. Associations of SNPs with 10 Year OS

In the training set, rs3918262-*MMP9*, rs7692791-*VEGFR2*, and rs2034967-*VEGFR2* were significantly associated with 10 year OS (Table 3). Some significant associations were observed in the replication set.

## 3. Discussion

Identification of predictive or prognostic immunogenetic markers is mandatory for a more complete understanding of the biomolecular mechanisms connecting RT and the immune response. The relationship between RT and the host immune response is also attracting new interest due to new immunotherapeutic treatments.

To address the clinical utility of the host immunogenetic biomarker in PCa, we developed a retrospective immunogenetics study of locally advanced PCa patients homogeneously treated with RT. The primary aim was to define 10 year biomarkers, while the secondary aims were defining biomarkers for 10 year OS, and analyzing associations between previously-defined prognostic biomarkers and 5 year BCR. Surprisingly, among the 447 analyzed SNPs, the only new potential immunogenetic variant of 10 year BCR was rs4143815-*PDL1*. Interestingly, it was significantly associated with 5 year BCR.

PDL1 is expressed in 20–50% of human cancers [9], including PCa [10]. PDL1 negatively regulates the immune response blocking the activation and proliferation of T cells expressing PD1 on their surface, playing a key role in the so-called cancer-immunity cycle, the mechanisms exploited by the immune system to effectively kill cancer cells [11]. PDL1 is also expressed in some immune cells (T and B lymphocytes and dendritic cells [12]), strongly highlighting its direct involvement in immune system activity and modulation. This has led to the development of cancer immunotherapies addressing PD1–PDL1 interaction, like nivolumab, pembrolizumab, durvalumab, and atezolizumab, which have been already integrated into the treatment of some solid tumors as advanced renal cell carcinoma, non small cell lung cancer (NSCLC), bladder cancer, and melanoma.

Usually, PCa is considered as an immunologically “cold” cancer. This assumption renders the immune therapy unconceivable in this clinical setting. However, an increasing amount of preclinical and clinical data have demonstrated the existence of immune mediators in PCa tissue, which increases the importance of investigations into the effect of the immune system on the definition of RT response. To go one step further, the definition of the potential role of immune therapy in PCa, and in combination with traditional treatments as RT, represents a new challenge [13,14,15]. Accordingly, many ongoing clinical trials are analyzing the potentialities of PD1/PDL1 inhibitors in PCa (clinivaltrials.gov) [16,17,18].

The rs4143815-*PDL1* has been previously proposed as a potential biomarker of gastric adenocarcinoma risk [19] and of NSCLC risk [20], and as a potential prognostic biomarker of OS in patients affected by NSCLC, lung adenocarcinoma, and squamous cell carcinoma who underwent curative surgical resection [21,22]. This SNP is located in the 3′UTR of PDL1 in the miR-570 binding site, modulating its expression [19]. Moreover, rs4143815-*PDL1* is linked with rs10815225-*PDL1*, a promoter variant involved PDL1 expression regulation [23]. Expression control of PDL1 plays a central clinical role, since its overexpression is a bad prognostic marker. It is correlated with tumor size, lymph node involvement, and patient prognosis [24,25,26].

Data from our study demonstrated that rs4143815-*PDL1* can be considered as a new prognostic biomarker for 10 year BCR in locally advanced PCa after RT. This SNP was significantly associated with 5 year BCR in both sets of patients.

Some issues are worth discussing. Usually, 5 year biomarkers are investigated and proposed to be integrated into clinical practice. This approach allows the prediction of most events, but not all. In accordance with the literature, most events in our study fell within 5 years from the end of RT (82.93% in the training set and 78.12% in the replication set). However, a non-negligible percentage of patient relapses occurred in the period spanning from 5 to 10 years (14.63% in the training set and 21.88% in the replication set), supporting the interest in biomarkers calculated over a longer period of time (Table S1).

This study was performed on germline DNA, where through a minimally invasive procedure (a blood drawn or a saliva sample), it is possible to obtain a biomarker that potentially could predict the risk of relapse in the following 10 years.

This study was performed with a pathway candidate approach based on a literature analysis. At present, other approaches are preferred, such as NGS studies. NGS clearly represents the future. Nonetheless, these analyses are still very expensive, requiring thousands of patients, and bioinformatics needs to evolve to face all the challenges associated with this approach. The design of our study, based on a pathway candidate approach, was supposed to give reliable and solid results that could be easily implemented in PCa management. Indeed, these results were obtained using well-balanced discovery and replication sets. Moreover, a solid statistical approach was applied. Multivariate analyses were performed using clinical parameters (i.e., PSA levels at diagnosis, age, Gleason score, hormone therapy, and tumor grade) significantly associated with 10 year BCR in accordance with the literature. In the discovery set, the FDR was applied with a strict cut-off (*q*-value < 0.15) to overcome the problem related to multiple testing.

The most significant limitation of this study is the relatively small number of patients included in the replication set, which reduces the statistical power of this analysis. Another point that should be addressed is that we did not analyze samples from tumor tissues. The concomitant analysis of germline and somatic DNA, and of protein expression, could clearly shed light on the clinical role of PDL1 in PCa. In light of this, it is feasible that a signature that considers not only genetic information, but also other “-omics” data and clinical parameters known to play roles in determining patients’ prognosis, could be the key to completely unravelling this issue. A wider approach like this could pave the way to a real clinical improvement in PCa treatment.

## 4. Materials and Methods

### 4.1. Patient Cohorts and Treatment

Between 2003 and 2008 at the CRO National Cancer Institute, Aviano (Italy), 549 locally advanced PCa patients were enrolled. All underwent a primary RT based regimen, with or without HT. The eligibility criteria were a histologically-confirmed diagnosis of locally advanced primary PCa, Caucasian ethnicity, age ≥ 18 years, a performance status (according to ECOG) of 0–2, and a life expectancy longer than 6 months. The exclusion criteria were no local disease control, impaired liver and renal function, comorbidities that rendered RT not possible, and relevant cardiovascular diseases. More details about patients’ treatment and eligibility criteria have already been published in [27].

According to the study’s purposes, patients were split into a discovery set (*n* = 418) and a replication set (*n* = 131), both well-balanced for clinical and pathological parameters (Table 1).

All patients signed a written informed consent for research purposes before entering this study, and all procedures were reviewed and approved by the Institutional Review Board of the CRO National Cancer Institute (11 May 2011; n° CRO-2011-18).

### 4.2. SNPs Selection

For this study, 576 SNPs localized in 77 genes related to immune system activity and analyzable with the BeadXpress platform (Illumina, Inc., San Diego, CA, USA) were defined (Figure S1).

Genes connected with the immune system and cancer were selected with a literature analysis. The blocks of SNPs with strong linkage disequilibrium (r^2^ ≥ 0.80) and MAF ≥ 0.05 in the Caucasian population were defined, and their genomic coordinates were analyzed with Haploregv2 software. Polymorphisms were prioritized according to their biological effect reported in the literature or predicted according to the SNPinfo web server (https://snpinfo.niehs.nih.gov/).

Polymorphisms with a high final score (≥0.7) and optimal designability (=1) according to the Illumina assay design tool were selected.

### 4.3. SNP Genotyping

The genomic DNA of PCa patients was extracted from peripheral blood samples using the automated extractor BioRobot EZ1, in association with the “EZ1 DNA Blood Kit 350μl” (Qiagen SPA, Milano, Italy), and stored at +4 °C until the time of this study.

Genomic DNA samples in the training set were analyzed using the Illumina BeadXpress platform (Illumina, Inc., San Diego, CA, USA). Fluorescence was detected with VeraScan software (version 2.0, Illumina, Inc., San Diego, CA, USA). GenomeStudio software 2010 (Illumina Inc., San Diego, CA, USA) was applied for genotype clustering, using a SNP call-threshold of 0.25 (on a scale of 0–1). Clusters were visually inspected and manually reviewed to ensure high quality data.

Only SNPs that showed significant results in the training set were analyzed in the replication set. *SMAD3*-rs7162912, *PDL1*-rs4143815, *SMAD2*-rs4940086, *VEGFR2*-rs12498529, *IL2RB*-rs84460, *SMAD3*-rs9302242, and *PDL1*-rs1411262, defined using pre-designed TaqMan SNP genotyping assays with the Applera TaqMan Universal Master mix, were used on ABI 7900HT (AB Applied Biosystem, Foster City, CA, USA). *STAT1*-rs16824035 was determined using Sanger sequencing (Biotage, Uppsala, Sweden), performing PCR amplifications in an Eppendorf Mastercycler gradient with TaqGold DNA polymerase (AB Applied Biosystem, Warrington, UK). *VEGFR2*-rs2034967, *miR641*-rs11880261, *VEGFR2*-rs7692791, and *MMP9*-rs3918262 were characterized using an allele-specific PCR with KASPar (KASP) reagents (LGC Genomics, Hoddesdon, United Kingdom). All of these analyses were performed according to the manufacturers’ instructions, including negative and positive controls.

### 4.4. Statistics: Identification of New Prognostic Biomarkers of BCR and OS

**Figure 2:**
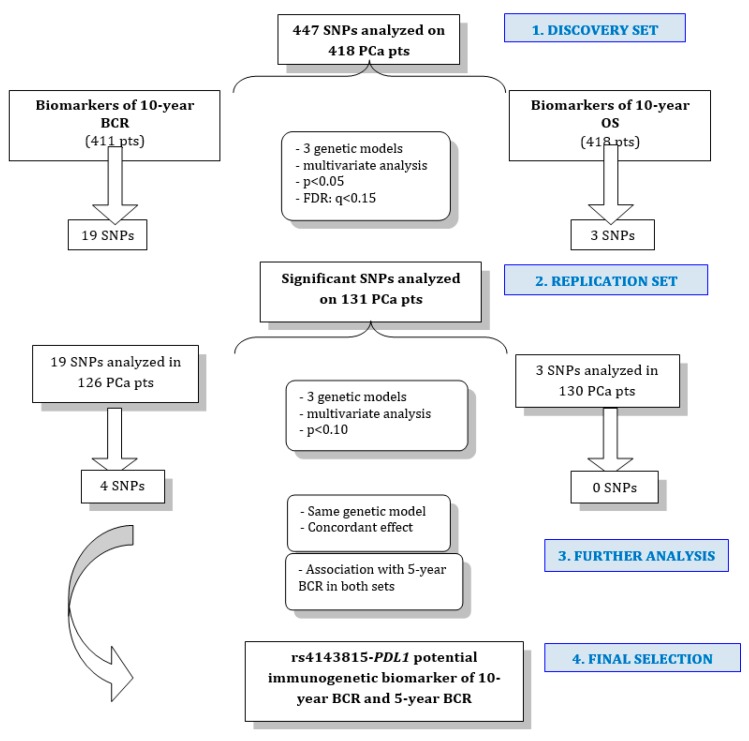
Study design. Firstly, analyses were performed on a discovery set and secondly, in a replication set. At the end of the study, rs4143815-PDL1 arose as a new potential immunogenetic biomarker of 10 year and 5 year BCR in locally advanced prostate cancer. Abbreviations: PCa, prostate cancer; BCR: biochemical recurrence; OS: overall survival; pts: patients.

The primary aim of this study was the identification of new potential biomarkers of 10 year BCR. Time at risk for BCR was defined as the interval time between the end of RT and an increase of PSA ≥ 2 ng/mL above the serum lowest level. The first secondary aim was the definition of new potential biomarkers of 10 year OS. This analysis was performed considering the interval time from the date of diagnosis to the date of death or last known follow-up, whichever came first (Figure 2). The other secondary aim was to determine whether replicated biomarkers were significantly associated with 5 year BCR.

In the training set, a multivariate Cox proportional hazard analysis was applied to test the associations between 10 year BCR and Gleason score, serum PSA level at diagnosis, age at diagnosis, TNM stage, D’Amico risk classes, and stage. Clinical parameters which featured significantly in this analysis were then used as covariates for both BCR and OS.

In the training set, associations between SNPs and 10 year BCR and 10 year OS were tested considering dominant, recessive, and additive genetic models. The best one was selected according to the Wald test. Hazard ratios (HRs) and 95% confidence intervals (95% CIs) were computed through a multivariate Cox proportional hazard model. False discovery rate (FDR) analysis was performed to reduce the number of false positive results. Consequently, significant SNPs were defined considering both the *p*-value (<0.05) and q-value (<0.15).

Only SNPs featuring significantly in the training set results for 10 year BCR and 10 year OS were analyzed in the replication set, testing the same genetic model that emerged in the training set. Prognostic markers were those SNPs which featured significantly in both groups and showed a concordant genetic effect (both HR< or >1). Replicated SNPs were tested for 5 year BCR in both sets of patients. Kaplan–Meier curves with the log-rank test were used to visualise the validated results. All the statistical analyses were performed using ‘R’ statistical package version 3.4.3.

## 5. Conclusions

This study defined rs4143815-*PDL1* as a new potential immunogenetic biomarker of BCR in locally advanced PCa patients undergoing RT, giving new insights into the correlation between RT and the immune system. If validated in prospective studies, it could be potentially implemented into clinical practice not only to stratify patients according to their risk of relapse, but also to potentially define those eligible for new immune therapies.

## Figures and Tables

**Table 1:** Pathological characteristics of analyzed prostate cancer patients. Data from the training set, replication set, and combined datasets are reported. Statistical analyses were performed to evaluate whether training set and replication set were homogeneous. The results are reported here. Abbreviations: RT: radiotherapy; BCR: biochemical recurrence; OS: overall survival.

Characteristics	*N*	Training Set	Replication Set	Combined Datasets	Test Statistic
		*N* = 418	*N* = 131	*N* = 549	
**Basal parameters**					
**Age**		69.8 ± 5.6	70.6 ± 4.8	70.0 ± 5.5	
TNM Grade	548				χ^2^_4_ = 3, *p*-value = 0.56 ^1^
0		0.01 ^3^⁄_418_	0.00 ^0^⁄_130_	0.01 ^3^⁄_548_	
1		0.08 ^32^⁄_418_	0.11 ^14^⁄_130_	0.08 ^46^⁄_548_	
2		0.59 ^248^⁄_418_	0.56 ^73^⁄_130_	0.59 ^321^⁄_548_	
3		0.32 ^134^⁄_418_	0.32 ^42^⁄_130_	0.32 ^176^⁄_548_	
4		0.00 ^1^⁄_418_	0.01 ^1^⁄_130_	0.00 ^2^⁄_548_	
Stage	549				χ^2^_3_ = 6.8, *p*-value = 0.079 ^1^
A		0.02 ^9^⁄_418_	0.02 ^2^⁄_131_	0.02 ^11^⁄_549_	
B		0.41 ^173^⁄_418_	0.53 ^69^⁄_131_	0.44 ^242^⁄_549_	
C		0.55 ^229^⁄_418_	0.46 ^60^⁄_131_	0.53 ^289^⁄_549_	
D		0.02 ^7^⁄_418_	0.00 ^0^⁄_131_	0.01 ^7^⁄_549_	
Gleason score	549				χ^2^_2_ = 0.69, *p*-value = 0.71 ^1^
2–6		0.56 ^235^⁄_418_	0.54 ^71^⁄_131_	0.56 ^306^⁄_549_	
7		0.23 ^97^⁄_418_	0.27 ^35^⁄_131_	0.24 ^132^⁄_549_	
8–10		0.21 ^86^⁄_418_	0.19 ^25^⁄_131_	0.20 ^111^⁄_549_	
D’Amico	549				χ^2^_2_ = 2.6, *p*-value = 0.28 ^1^
1		0.24 ^100^⁄_418_	0.31 ^40^⁄_131_	0.26 ^140^⁄_549_	
2		0.13 ^55^⁄_418_	0.14 ^18^⁄_131_	0.13 ^73^⁄_549_	
3		0.63 ^263^⁄_418_	0.56 ^73^⁄_131_	0.61 ^336^⁄_549_	
PSA at diagnosis (ng/mL)	549				χ^2^_2_ = 0.11, *p*-value = 0.95 ^1^
<4		0.06 ^27^⁄_418_	0.07 ^9^⁄_131_	0.07 ^36^⁄_549_	
>10		0.43 ^180^⁄_418_	0.44 ^58^⁄_131_	0.43 ^238^⁄_549_	
4–10		0.50 ^211^⁄_418_	0.49 ^64^⁄_131_	0.50 ^275^⁄_549_	
**Treatment**					
Hormone therapy	549	0.86 ^359^⁄_418_	0.87 ^114^⁄_131_	0.86 ^473^⁄_549_	χ^2^_1_ = 0.11, *p*-value = 0.74 ^1^
RT dose (cGy)	549				χ^2^_4_ = 4.3, *p*-value = 0.36 ^1^
6600		0.01 ^4^⁄_418_	0.00 ^0^⁄_131_	0.01 ^4^⁄_549_	
7000		0.09 ^36^⁄_418_	0.05 ^7^⁄_131_	0.08 ^43^⁄_549_	
7400		0.12 ^50^⁄_418_	0.11 ^14^⁄_131_	0.12 ^64^⁄_549_	
7600		0.74 ^311^⁄_418_	0.82 ^107^⁄_131_	0.76 ^418^⁄_549_	
8000		0.04 ^17^⁄_418_	0.02 ^3^⁄_131_	0.04 ^20^⁄_549_	
**Follow-up**					
BCR	549	0.19 ^80^⁄_418_	0.24 ^32^⁄_131_	0.20 ^112^⁄_549_	χ^2^_1_ = 1.7, *p*-value = 0.19 ^1^
Follow-up BCR	538	22 (43–70)	21 (46–68)	22 (45–70)	*F*_1 536_ = 0.11, *p*-value = 0.74 ^2^
		47 ± 30	46 ± 31	47 ± 30	
Death	549	0.18 ^76^⁄_418_	0.19 ^25^⁄_131_	0.18 ^101^⁄_549_	χ^2^_1_ = 0.05, *p*-value = 0.82 ^1^
Follow-up OS	549	45 (68–94)	43 (65–90)	44 (68–94)	*F*_1 547_ = 1.2, *p*-value = 0.26 ^2^
		70 ± 31	66 ± 32	69 ± 32	

*a*,*b*,*c* represents the lower quartile. *a* is the the median. *b* is the upper quartile. *c* represents continuous variables. *x* ± *s* represents X ± 1 SD. *N* is the number of non-missing values. The following tests were used: ^1^ Pearson test; ^2^ Wilcoxon test.

**Table 2:** *Association between SNPs and BCR*. Hazard ratio (HR) and 95% confidence interval (95% CI) in the discovery (*n* = 411) and replication (126) sets according to gene polymorphisms (SNPs) are listed. Only SNPs that were significant (*p* < 0.05 and *q* < 0.15) in the discovery set are reported. Results obtained in the replication cohort are listed. The rs4143815-*PDL1* is shown in bold: it presents the same predictive effect in both groups (HR < 1) and is associated with BCR in the replication set with a trend.

Gene	SNP	Base Change	M	Discovery Cohort (*n* = 411) *			Replication Cohort (*n* = 126) *	
				HR (95% CI)	*p*-Value	*q*-Value	HR (95% CI)	*p*-Value
*IL2RB*	rs84460	C/T	R	2.79 (1.34–5.81)	0.006	0.121	0.96 (0.12–7.42)	0.97
*SMAD3*	rs7162912	G/T	A	2.04 (1.43–2.92)	0.0001	0.001	0.80 (0.44–1.45)	0.46
*FOXO3*	rs7762395	A/G	A	1.91 (1.26–2.89)	0.002	0.121	0.74 (0.31–1.79)	0.51
*FOXO3*	rs2153960	C/T	A	1.83 (1.27–2.64)	0.001	0.101	1.11 (0.63–1.98)	0.71
*SMAD3*	rs9302242	A/G	R	2.03 (1.24–3.32)	0.005	0.128	1.81 (0.82–4.02)	0.14
*IL4R*	rs1805011	A/C	D	2.28 (1.32–3.92)	0.003	0.121	1.32 (0.51–3.42)	0.57
*IL4R*	rs3024586	A/G	D	2.56 (1.36–4.80)	0.003	0.121	0.56 (0.15–2.15)	0.40
*CCL5*	rs2280789	C/T	R	7.93 (2.31–27.15)	0.001	0.101	0.70 (0.09–5.74)	0.74
*TLR2*	rs3804099	C/T	A	1.71 (1.24–2.36)	0.001	0.109	1.39 (0.74–2.61)	0.30
*PDL1*	rs1411262	A/G	A	1.6 (1.15–2.28)	0.005	0.123	0.48 (0.24–0.97)	0.04
*PDL1*	rs10125854	A/G	D	3.01 (1.66–5.45)	0.0003	0.065	0.54 (0.17–1.70)	0.30
***PDL1***	**rs4143815**	**C/G**	**A**	**0.58 (0.41–0.83)**	**0.003**	**0.117**	**0.52 (0.26–1.04)**	**0.06**
*SMAD2*	rs1792666	A/T	D	2.09 (1.25–3.50)	0.005	0.128	2.99 (1.10–8.12)	0.03
*SMAD2*	rs4940086	C/T	A	1.65 (1.18–2.32)	0.004	0.117	0.29 (0.14–0.59)	0.0007
*STAT1*	rs16824035	C/T	R	3.97 (1.68–9.39)	0.002	0.117	0 (0; inf)	1.00
*VEGFR2*	rs12498529	A/T	R	4.26 (1.64–11.06)	0.003	0.121	1.45 (0.31–6.80)	0.64
*VEGFR2*	rs2034965	A/G	A	1.60 (1.14–2.27)	0.007	0.154	0.74 (0.41–1.33)	0.32
*VEGFR2*	rs4576072	C/T	R	4.67 (1.59–13.74)	0.005	0.128	1.76 (0.38–8.22)	0.47
*AKT2/miR641*	rs11880261	C/T	A	0.64 (0.44–0.92)	0.02	0.128	1.33 (0.74–2.36)	0.34

* Adjusted for PSA level at diagnosis, age, Gleason score, hormone therapy, and tumor grade. Abbreviations: M: genetic model; R: recessive, A: additive; D: dominant; HR: hazard ratio; 95% CI: 95% confidence interval. All these SNPs were analyzed in the replication set. At the end of the analysis, according to the additive model, only rs4143815-*PDL1* wassignificantly associated with 10 year BCR with a borderline significance (training set: *p* = 0.003, HR (95% CI) = 0.58 (0.41–0.83); replication set: *p* = 0.063, HR (95% CI) = 0.51 (0.25–1.00)).

**Table 3:** *Association between SNPs and OS*. Hazard ratios (HR) and 95% confidence intervals (95% CI) in the discovery (*n* = 418) and replication (130) sets according to gene polymorphisms (SNPs) are listed. Only SNPs that were significant (*p* < 0.05 and *q* < 0.15) in the discovery cohort are reported. Results obtained in the replication cohort are listed.

Gene	SNP	Base Change		Discovery Cohort (*n* = 418) *			Replication Cohort (*n* = 130) *	
			M	HR (95% CI)	*p*-Value	*q*-Value	HR (95% CI)	*p*-Value
*MMP9*	rs3918262	A/G	R	4.31 (1.81–10.26)	0.001	0.14	0 (0–Inf)	1.00
*VEGFR2*	rs7692791	C/T	A	1.82 (1.31–2.53)	0.0003	0.14	1.09 (0.58–2.03)	0.79
*VEGFR2*	rs2034967	C/T	D	0.45 (0.28–0.72)	0.00000008	0.14	0.68 (0.30–1.56)	0.36

* Adjusted for PSA level at diagnosis, age, Gleason score, hormone therapy, and tumor grade. Abbreviations: M: genetic model; R: recessive, A: additive; D: dominant; HR: hazard ratio; 95% CI: 95% confidence interval.

## Supplementary Materials

Supplementary materials can be found at https://www.mdpi.com/1422-0067/20/9/2082/s1.

## References

[1] Xie W., Regan M.M., Buyse M., Halabi S., Kantoff P.W., Sartor O., Soule H., Clarke N.W., Collette L., Dignam J.J. (2017). Metastasis-Free Survival Is a Strong Surrogate of Overall Survival in Localized Prostate Cancer. J. Clin. Oncol..

[2] Williams S. (2018). Surrogate endpoints in early prostate cancer research. Transl. Androl. Urol..

[3] De Langhe S., De Ruyck K., Ost P., Fonteyne V., Werbrouck J., De Meerleer G., De Neve W., Thierens H. (2013). Acute radiation-induced nocturia in prostate cancer patients is associated with pretreatment symptoms, radical prostatectomy, and genetic markers in the TGFβ1 gene. Int. J. Radiat. Oncol. Biol. Phys..

[4] Langsenlehner T., Thurner E.M., Renner W., Gerger A., Kapp K.S., Langsenlehner U. (2014). Association of genetic variants in VEGF-A with clinical recurrence in prostate cancer patients treated with definitive radiotherapy. Strahlenther. Onkol..

[5] Renner W., Langsenlehner U., Krenn-Pilko S., Eder P., Langsenlehner T. (2017). BCL2 genotypes and prostate cancer survival. Strahlenther. Onkol..

[6] Schoenfeld J.D., Margalit D.N., Kasperzyk J.L., Shui I.M., Rider J.R., Epstein M.M., Meisner A., Kenfield S.A., Martin N.E., Nguyen P.L. (2013). A single nucleotide polymorphism in inflammatory gene RNASEL predicts outcome after radiation therapy for localized prostate cancer. Clin. Cancer Res..

[7] Cushman T.R., Caetano M.S., Welsh J.W., Verma V. (2018). Overview of ongoing clinical trials investigating combined radiotherapy and immunotherapy. Immunotherapy.

[8] Eze C., Manapov F., Gratzke C., Schmidt-Hegemann N.S., Jung A., Kirchner T., Heinemann V., Stief C.G., Belka C., Boeck S. (2018). Concurrent radiotherapy and nivolumab in metachronous metastatic primary adenosquamous-cell carcinoma of the prostate. Eur. J. Cancer.

[9] Herbst R.S., Gordon M.S., Fine G.D., Sosman J.A., Soria J.C., Powderly O.H.J.D., Burris H.A., Mokatrin A., Kowanetz M., Leabman M. (2013). A study of MPDL3280A, an engineered PD-L1 antibody in patients with locally advanced or metastatic tumors. JCO.

[10] Calagua C., Russo J., Sun Y., Schaefer R., Lis R., Zhang Z., Mahoney K., Bubley G.J., Loda M., Taplin M.E. (2017). Expression of PD-L1 in Hormone-naïve and Treated Prostate Cancer Patients Receiving Neoadjuvant Abiraterone Acetate plus Prednisone and Leuprolide. Clin. Cancer Res..

[11] Chen D.S., Mellman I. (2013). Oncology meets immunology: the cancer-immunity cycle. Immunity.

[12] Zou W., Chen L. (2008). Inhibitory B7-family molecules in the tumour microenvironment. Nat. Rev. Immunol..

[13] Kwek S.S., Cha E., Fong L. (2012). Unmasking the immune recognition of prostate cancer with CTLA4 blockade. Nat. Rev. Cancer.

[14] Modena A., Ciccarese C., Iacovelli R., Brunelli M., Montironi R., Fiorentino M., Tortora G., Massari F. (2016). Immune Checkpoint Inhibitors and Prostate Cancer: A New Frontier?. Oncol. Rev..

[15] Montironi R., Santoni M., Sotte V., Cheng L., Lopez-Beltran A., Massari F., Matrana M.R., Moch H., Berardi R., Scarpelli M. (2016). Emerging Immunotargets and Immunotherapies in Prostate Cancer. Curr. Drug. Targets.

[16] Brahmer J.R., Drake C.G., Wollner I., Powderly J.D., Picus J., Sharfman W.H., Stankevich E., Pons A., Salay T.M., McMiller T.L. (2010). Phase I study of single-agent anti-programmed death-1 (MDX-1106) in refractory solid tumors: safety, clinical activity, pharmacodynamics, and immunologic correlates. J. Clin. Oncol..

[17] Topalian S.L., Hodi F.S., Brahmer J.R., Gettinger S.N., Smith D.C., McDermott D.F., Powderly J.D., Carvajal R.D., Sosman J.A., Atkins M.B. (2012). Safety, activity, and immune correlates of anti-PD-1 antibody in cancer. N. Engl. J. Med..

[18] Taube J.M., Klein A., Brahmer J.R., Xu H., Pan X., Kim J.H., Chen L., Pardoll D.M., Topalian S.L., Anders R.A. (2014). Association of PD-1, PD-1 ligands, and other features of the tumor immune microenvironment with response to anti-PD-1 therapy. Clin. Cancer Res..

[19] Wang W., Li F., Mao Y., Zhou H., Sun J., Li R., Liu C., Chen W., Hua D., Zhang X. (2013). A miR-570 binding site polymorphism in the B7-H1 gene is associated with the risk of gastric adenocarcinoma. Hum. Genet..

[20] Du W., Zhu J., Chen Y., Zeng Y., Shen D., Zhang N., Ning W., Liu Z., Huang J.A. (2017). Variant SNPs at the microRNA complementary site in the B7-H1 3′-untranslated region increase the risk of non-small cell lung cancer. Mol. Med. Rep..

[21] Yeo M.K., Choi S.Y., Seong I.O., Suh K.S., Kim J.M., Kim K.H. (2017). Association of PD-L1 expression and PD-L1 gene polymorphism with poor prognosis in lung adenocarcinoma and squamous cell carcinoma. Hum. Pathol..

[22] Lee S.Y., Jung D.K., Choi J.E., Jin C.C., Hong M.J., Do S.K., Kang H.G., Lee W.K., Seok Y., Lee E.B. (2017). Functional polymorphisms in PD-L1 gene are associated with the prognosis of patients with early stage non-small cell lung cancer. Gene.

[23] Tao L.H., Zhou X.R., Li F.C., Chen Q., Meng F.Y., Mao Y., Li R., Hua D., Zhang H.J., Wang W.P. (2017). A polymorphism in the promoter region of PD-L1 serves as a binding-site for SP1 and is associated with PD-L1 overexpression and increased occurrence of gastric cancer. Cancer Immunol. Immunother..

[24] Wang W., Sun J., Li F., Li R., Gu Y., Liu C., Yang P., Zhu M., Chen L., Tian W. (2012). A frequent somatic mutation in CD274 3′-UTR leads to protein over-expression in gastric cancer by disrupting miR-570 binding. Hum. Mutat..

[25] Harada K., Ferdous T., Ueyama Y. (2018). PD-L1 expression in malignant salivary gland tumors. BMC Cancer.

[26] Zhao Y.J., Sun W.P., Peng J.H., Deng Y.X., Fang Y.J., Huang J., Zhang H., Wan D., Lin J., Pan Z. (2018). Programmed death-ligand 1 expression correlates with diminished CD8+ T cell infiltration and predicts poor prognosis in anal squamous cell carcinoma patients. Cancer Manag. Res..

[27] Zanusso C., Bortolus R., Dreussi E., Polesel J., Montico M., Cecchin E., Gagno S., Rizzolio F., Arcicasa M., Novara G. (2017). Impact of DNA repair gene polymorphisms on the risk of biochemical recurrence after radiotherapy and overall survival in prostate cancer. Oncotarget.

