# Supplementary material for: rs4143815-PDL1, a New Potential Immunogenetic Biomarker of Biochemical Recurrence in Locally Advanced Prostate Cancer after Radiotherapy
Source: Int J Mol Sci. 2019 Apr 27;20(9):2082. doi: 10.3390/ijms20092082 (PMC6539607; doi:10.3390/ijms20092082)
Supplement: Supplementary file 1 [file ijms-20-02082-s001.pdf]

rs4143815-PDL1, a New Potential Immunogenetic Biomarker of Biochemical Recurrence in Locally Advanced Prostate Cancer after Radiotherapy

Supplementary table 1. Time-dependent distributions of BCR in the discovery set and in the replication set. All events are reported.

Period (months)	Discovery set			Replication set		
	n	%		n	%	
<12	16	19.5	BCR < 60 months: 68 (82.93%)	5	15.6	BCR < 60 months: 25 (78.12%)
≥12 <24	20	24.4		7	21.9	
≥24 <36	15	18.3		3	9.4	
≥36 <48	12	14.6		7	21.9	
≥48 <60	5	6.1		3	9.4	
≥60 <72	7	8.5	BCR 60-120 months: 12 (14.63%)	2	6.3	BCR 60-120 months: 7 (21.88%)
≥72 <84	1	1.2		3	9.4	
≥84 <96	0	0.0		1	3.1	
≥96 <108	2	2.4		0	0.0	
≥108 <120	2	2.4		1	3.1	
≥120	2	2.4	BCR ≥120 months: 2 (2.44%)	0	0.0	BCR ≥120 months: 0 (0.0%)

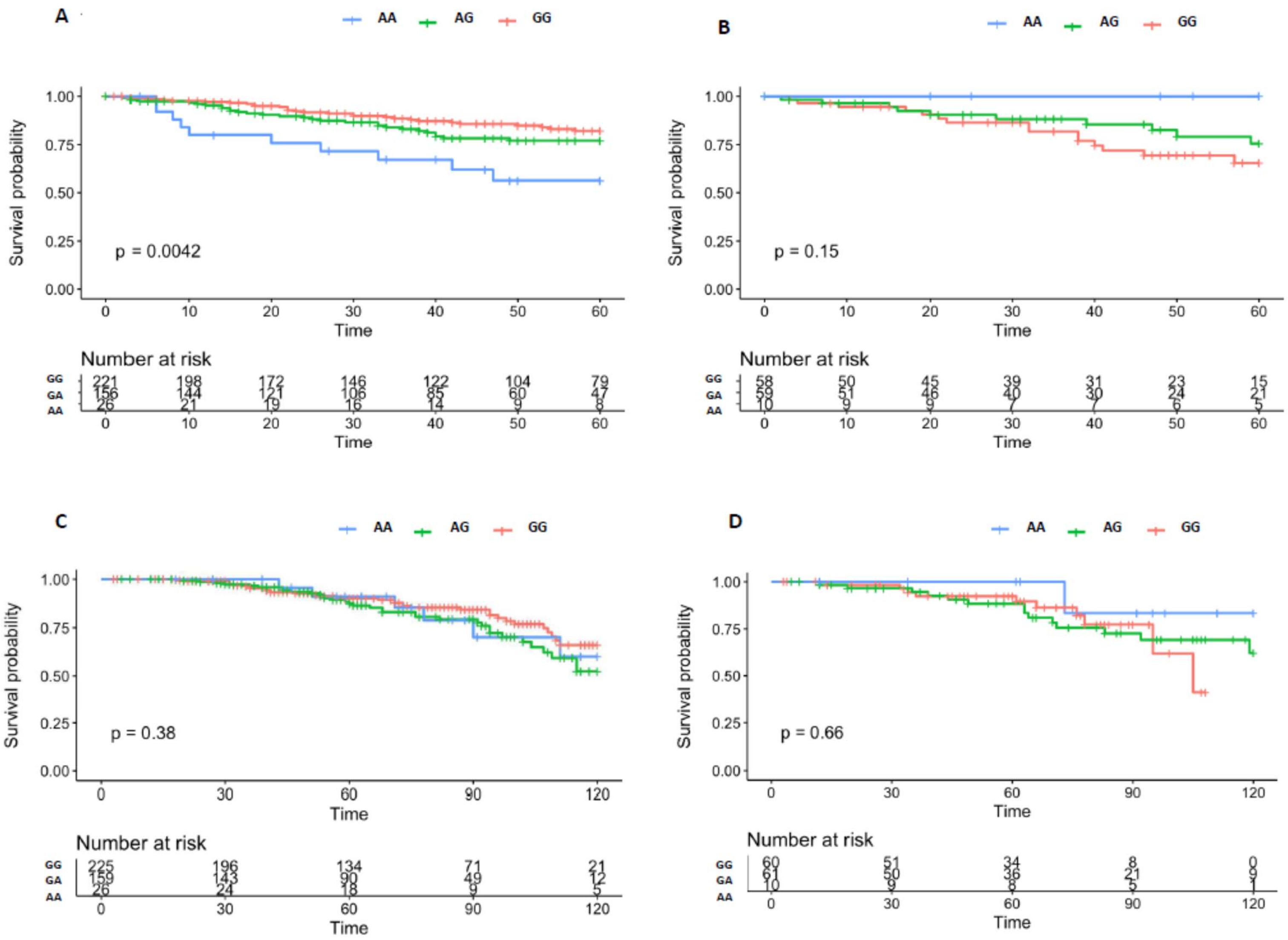

Supplementary Figure 1. Kaplan-Meier curves of 5-year BCR accoding to rs1411262-*PDL1* in the training set (A) and in the replication set (B) and Kaplan-Meier curves of 10-year OS according to rs1411262-*PDL1* in the training set (C) and in the replication set (D).
